# Peripheral Edema Occurring during Treatment with Risperidone Combined with Citalopram

**DOI:** 10.1155/2012/540732

**Published:** 2012-11-01

**Authors:** Seyed Hamzeh Hosseini, Amirhossein Ahmadi

**Affiliations:** ^1^Psychiatry and Behavioral Sciences Research Center and Department of Psychiatry, Faculty of Medicine, Mazandaran University of Medical Sciences, Sari 48175-861, Iran; ^2^Pharmaceutical Sciences Research Center, Faculty of Pharmacy, Mazandaran University of Medical Sciences, Sari 48175-861, Iran

## Abstract

An 80-year-old female presented with symptoms of depression, worthlessness, hopelessness, loss of energy, insomnia, impatience, and forgetfulness associated with persecutory delusion that had begun about one year before her visit. She was diagnosed with major depression with psychotic signs and began treatment with risperidone (2 mg/night) and citalopram (20 mg/day). After 20 days, she returned and reported partial improvement in her symptoms, although she had developed severe swelling of the hands and feet. The results of liver and renal function tests and rheumatologic tests were found to be within normal limits. Risperidone was discontinued for a week, and the swelling resolved completely. Risperidone was then administered again, and the swelling returned so that the patient had to discontinue taking the drug. The reappearance of edema on rechallenge is strong evidence implicating risperidone as the cause of the swelling.

## 1. Background 

Risperidone, a benzisoxazole derivative, is a novel antipsychotic agent that combines potent serotonin (5-hydroxytryptamine, 5-HT2) and dopamine (D2) receptor antagonism [1]. Risperidone is a widely prescribed atypical antipsychotic agent that seems to be effective in behavioral problems, including hyperactivity, irritability, aggressiveness, self-injurious behavior, and stereotypies [2]. Several studies in adolescents demonstrate the effectiveness of risperidone for treating disruptive and aggressive behaviors [3]. Weight gain and sedation are among the most commonly reported adverse effects [3]; however, edema was recently included as an adverse effect of risperidone in the British National Formulary (2002). 

Angioedema is characterized by edema of the deep dermal and subcutaneous tissues. It is considered to occur secondary to the release of proinflammatory modulators, particularly through an immunoglobulin E (IgE)-mediated mechanism (type I hypersensitivity reaction). It is thought that the antipsychotic or its metabolite combines with IgE on the surface of the cutaneous mast cells, which leads to the release of vasoactive substances such as histamine, leukotrienes, and prostaglandin, leading to vascular dilatation and increased vascular permeability resulting in edema [4]. However, other mechanisms have also been suggested [5].

To the best of our knowledge, there have been 9 cases of edema associated with risperidone. In 6 of these cases, risperidone was used in combination with other psychotropic agents, including valproate, benzodiazepines, and/or dopamine receptor antagonists. The complex interaction between risperidone and other psychotropic drugs may have contributed to the edema reported in these cases [6–10]. Among them, one case documented an allergic reaction to risperidone resulting in edema [11]. Consequently, only the last 3 cases reported the occurrence of edema subsequent to oral risperidone administered alone [5, 12]. Nevertheless, edema occurring during treatment with risperidone combined with citalopram, an antidepressant agent, has not been reported previously. Here, we report a case of edema with risperidone following citalopram medication.

## 2. Case Presentation

An 80-year-old female presented with symptoms of depression, worthlessness, hopelessness, loss of energy, insomnia, impatience, and forgetfulness associated with self-injurious behavior that had begun about a year before her visit. She was diagnosed with major depression with psychotic signs and began treatment with risperidone (2 mg/night) and citalopram (20 mg/day), half the dose was taken in the morning and the other half was taken at night, (10 mg at morning and 10 mg at night). After 20 days, she returned and reported a partial improvement in her symptoms, although she had developed severe swelling of hands and feet from the wrist to the tip of her fingers or tip of her toes. Complete examinations including liver function tests, renal function tests, and rheumatological tests were performed. The results were found to be within normal limits, and the patient had no physical problems. Subsequently, a diagnosis of risperidone-associated angioedema was made. Risperidone treatment was discontinued for a week, and the swelling resolved completely. Risperidone was then administered again, and the swelling returned so that the patient had to discontinue taking the drug. Eventually, the patient was administered citalopram and Seroquel. After changing the medication, no side effects were observed. 

## 3. Discussion

There are reports of peripheral edema associated with citalopram [13]. In June 8, 2012, as reported in a postmarketing study of peripheral edema among people who take citalopram hydrobromide, 13,424 people had reported side effects when taking citalopram hydrobromide. Among them, 214 people (1.59%) had peripheral edema [14]. However, in our case, the recurrence of edema during risperidone rechallenge is strong evidence implicating risperidone and suggesting that risperidone, and not citalopram, was the responsible agent. 

There have been a number of case reports of edema occurring in a dose-dependent manner with risperidone therapy [7, 15, 16]. In some of these cases, risperidone was used in combination with sodium valproate and/or benzodiazepines. In one case report, the edema disappeared when a diuretic was added to the risperidone therapy [6]. In the cases reported by Sanders and Lehrer and also Ravasia, the edema fully resolved when risperidone was either decreased or discontinued [7, 10]. In most cases, no abnormal findings were obtained in either the hematological or immunological examinations, and the exact mechanism by which risperidone caused the edema was not clear. 

There are several explanations that may account for the onset of edema secondary to risperidone therapy [17]. First, the action of risperidone on the *α*-receptors of the peripheral vascular system may have caused vasodilation and raised the hydrostatic pressure in the blood capillaries, which would result in edema [18]. Second, risperidone-induced 5-HT2 receptor blockade can potentially increase the levels of cyclic adenosine monophosphate, which relaxes vascular smooth muscle through the phosphorylation of myosin light chain kinase [19]. Third, dopaminergic blockade plays a role in producing edema by altering the renal regulation of fluid and electrolytes [20]. In addition, several other possible underlying mechanisms, such as allergic reactions, supersensitivity of receptors, and complex drug interactions, are postulated [21]. Conney and Nagy reported C4-C2 activation in a patient with previously low C1 inhibitor activity [22], and Terao et al. reported type I and type IV allergic reactions in another patient [11]. In another case, edema occurred when risperidone was reinstated after a drug-free period rather than during the first 4 months of treatment. It was hypothesized that this occurrence of edema may have been due to the supersensitivity of the *α*-receptors to risperidone, which may have occurred during the drug-free period. In addition, there is a “critical drug-free period” during which, if risperidone is reinstated, the side effects are exaggerated because of the supersensitivity of the neurons [12].

The recent increase in the number of cases of edema occurring during risperidone treatment indicates that this possible complication is not rare. The complication of edema induced by risperidone is usually overlooked by physicians unless the patients complain. A review of the literature showed that angioedema can occur at any time during therapy with risperidone. Physicians should be aware of this adverse effect of risperidone and, whenever it is suspected, should switch to an antipsychotic drug that has different pharmacodynamics, such as aripiprazole, which may decrease and suspend the edematous reaction. However, available data also suggest that the effect may be dose-dependent, and if no other alternative is available, a lower dose may be an alternative. In addition, depending on the severity of the symptoms, patients should be treated with supportive agents, such as a diuretic. Of course, it is better to not rechallenge with risperidone in the future, as our study shows that edema tends to recur.

Because a plausible mechanism of risperidone-induced edema remains unknown, further investigation is warranted to elucidate the characteristics, risk factors, dose dependence, and potential mechanisms of risperidone-associated edema as well as the perfect type of treatment. We hope this paper will be a warning to physicians who decide to prescribe risperidone.
